# Heider Balance—A Continuous Dynamics

**DOI:** 10.3390/e27080841

**Published:** 2025-08-08

**Authors:** Krzysztof Kułakowski

**Affiliations:** Faculty of Physics and Applied Computer Science, AGH University of Krakow, al. Mickiewicza 30, 30-059 Cracow, Poland; kulakowski@fis.agh.edu.pl

**Keywords:** social simulations, differential equations, cognitive dissonance

## Abstract

This paper is a short review on applications of non-linear dynamics in the concept of Heider balance, known also as structural balance. In all the papers listed here, the basic tools are ordinary differential equations. All papers pay attention to real social phenomena, which play the role of illustrations of the mathematical formalisms.

## 1. Introduction

The concept of social networks was introduced in 1934 by Jacob Moreno [1]. Various structures of friendly and hostile relations have accordingly been distinguished and represented by signed links [1] (pp. 104–107). Since then, analysis of structures of social networks has become a separate branch of mathematical sociology [2]. In the course of this research, the network topology is represented by a connectivity matrix, with the matrix element $x_{ij}$ equal to one or zero when a link between nodes *i* and *j* exists or not. With signed links, the description is more rich: the matrix elements can represent positive (friendly) or negative (hostile) relations. The present text is mostly devoted to the case where the variables are real numbers, which allows us to include the intensity of relations.

In its most popular version, the Heider balance is formulated as the specific state of a network with signed links. Links can be positive (friendly) or negative (hostile). A state is balanced if [3]:

- My friend’s friend is my friend;
- My friend’s enemy is my enemy;
- My enemy’s friend is my enemy;
- My enemy’s enemy is my friend.

The driving force of the evolution of the network towards a balanced state is a desire to avoid cognitive dissonance [4], which appears if the partition of neighbors into enemies and friends is not coherent. An example of this incoherence is when my enemy’s enemy is my enemy. Is she my friend because she hates my enemy, or is she my enemy as she is supposed to? To remove this unpleasant feeling, people modify their interpersonal relations: hostile to friendly or friendly to hostile.

**Figure 1:**
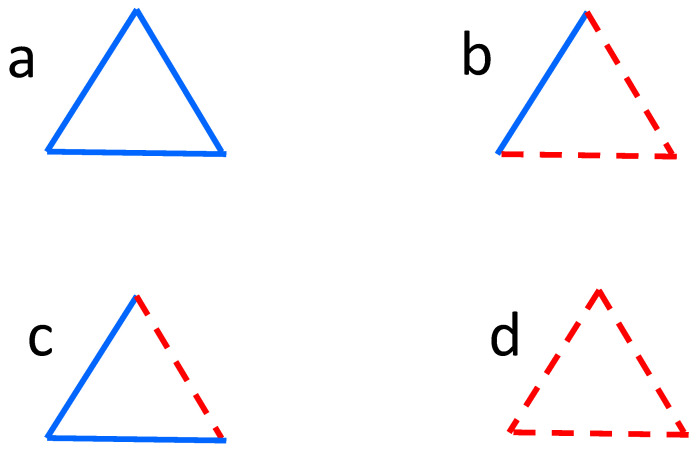
Four possible states of a triad of links, where blue continuous lines mean positive (friendly) relations, and red dashed lines mean negative (hostile) relations. The product of three relations within a triad is positive for balanced triads, as in (**a**,**b**), and negative for imbalanced triads, as in (**c**,**d**).

The description of the time evolution towards balance as the switching signs of two-valued links was well established long ago [5]; applications of continuous dynamics appeared later. The aim of the present text is to review attempts at the formulation of the Heider balance problem in terms of differential equations. Therefore, only a few notes are devoted to the case of two-valued links in the following. Most of the research in this regard can be summarized as applications of statistical mechanics, with the Ising model [6] as a point of departure. In this discrete version, spins $x_{ij}=\pm 1$ are assigned to all links. Further, all triads of mutually connected nodes ijk are qualified as balanced if $x_{ij}x_{jk}x_{ki}=+1$ and imbalanced otherwise; see Figure 1. The number of imbalanced triads plays the role of energy and is minimized within the usual Monte Carlo formalism [7]. To give a few examples, in [8] a mean-field approach is applied to investigate a phase transition, where the balanced state vanishes. Similarly in [9] a multiplex system is investigated, and a critical temperature is obtained with the aid of mean-field theory. The temperature where the system is switched from the paradise state (all links positive) to an imbalanced state is also investigated in [10]. There, the transition is discontinuous and irreversible. In all these cases, the simulated system evolves in the presence of thermal noise.

Basically, the postulated dynamics should drive the system to a balanced state. This condition should be refined, however, to allow for the so-called jammed states, stationary yet imbalanced. This option has been formalized by the so-called structure theorems [11]:

- First structure theorem [5]: A signed graph is balanced if and only if the set of nodes can be partitioned into two subsets so that every positive link joins nodes of the same subset and every negative link joins nodes of different subsets;
- Second structure theorem [12]: A signed graph is k-balanced for k≥2 if and only if the set of nodes can be partitioned into k subsets so that every positive link joins nodes of the same subset and every negative link joins nodes of different subsets.

For our purposes here, the most essential is the so-called fundamental structural balance theoretic hypothesis (FSBH) [11]: “signed networks move toward balance over time”. By balance, we admit states defined both in the first and in the second structure theorems. Later in this text, we will also admit limit cycles as a solution. It is worthwhile to note that the second structure theorem admits also states where the statement: “my enemy’s enemy is my friend” is not true. A simple example is the case k=3, that is, of three subsets, each consisting of three nodes, with positive links within each subset and negative links between nodes in different subsets [13]. As we will note later, this state is a stationary solution of some variant of the Heider dynamics.

This text can be seen as a continuation of a similar review by the same author 18 years ago [14]. The scope is narrowed: to be included here, a paper should analyze a continuous nonlinear equation of motion, and it should contain some relations to the social sciences. In addition to new material, some repetitions of [14] are found to be necessary, and some details are corrected. The perspective is at least updated.

## 2. The Compartmental Model

The equations of motion reported here have been introduced in [13,15]. There are two versions of this approach; what we report here is termed “Local Triad Dynamics”. Consider a complete graph with signed links. There are four types of triad, $\Delta_{k}$ with *k* = 0, 1, 2, or 3, where *k* is the number of negative links. Then *k* = 0 and 2 contribute to balanced states and *k* = 1 and 3 to imbalanced states. According to the FSBH, the system evolves to balance. The related probabilities are parameterized as follows: $\Delta_{1}\to \Delta_{0}$ with probability *p*, $\Delta_{1}\to \Delta_{2}$ with probability 1−p, and $\Delta_{3}\to \Delta_{2}$ with probability 1. It is clear that the concentration of positive links increases with *p*. With probabilistic considerations, differential equations of motion are obtained for the densities of all kinds of triad. This kind of system is called the “compartmental model” [16]. Denoting the density of friendly links by ρ and neglecting the correlations between different types of triad, the equation of motion for ρ can be written as

(1) $$\dot{\rho }=3\left(2p-1\right)\rho^{2}\left(1-\rho \right)+{\left(1-\rho \right)}^{3}$$

The analytical solution, including stable fixed points, is provided in [15] separately for p<1/2 and for p>1/2. In the former case, friendly and hostile links coexist: $\rho \left(t\to \infty \right)={\left[1+\sqrt{3(1-2p)}\right]}^{-1}$. Also, as all types of triads appear, the balanced state is not attained there. For the latter, ρ(t) tends to one; all links become friendly. This phase is called “paradise” [13].

For completeness, we should add that another type of evolution has also been provided in [13,15]: the so-called “Constrained Triad Dynamics”. There, modifications of links such that the number of imbalanced triads increases are forbidden. As a consequence, jammed states are possible, such as the one mentioned in the Introduction.

The mechanism of attaining balance has been illustrated in [13,15] with an example of international relations before World War I. That is, the outbreak was preceded by an evolution of relations until the balanced state was established, with Austria-Hungary, Germany, and Italy on one side, and Great Britain, Russia, and France on the other.

## 3. Gradual Relations

The next idea was to interpret the link variables as gradual relations. Instead of being just ±1, a link $x_{ij}$ between nodes *i* and *j* is characterized by a real number $-1\leq x_{ij}\leq +1$. In a complete graph of *N* nodes, each link belongs to N−2 triads. Each triad of nodes i,j,k contributes to the evolution of the link between nodes *i* and *j*: $x_{ij}$ increases if the product $x_{ik}x_{kj}$ is positive and decreases if the same product is negative. In addition, the rates of this evolution depend on these products. In summary, each link evolves according to its own equation of motion [17]

(2) $${\dot{x}}_{ij}=G\left(x_{ij}\right)\sum_{k\neq i,j}^{N-2}x_{ik}x_{kj}$$

where the factor $G\left(x\right)=1-x^{2}$ is introduced to keep *x* in the range [−1,+1].

Numerical solutions indicate that the set of Equation (2) generically drives the network to one of the $2^{N-1}$ balanced states. There, the variables $x_{ij}$ assume the values ±1. With the function G(x) chosen as above, the stability of the fixed points can be checked analytically because non-diagonal elements of the Jacobian vanish. The stability condition of the fixed point is then just that all diagonal elements of the Jacobian are negative. They are easily calculated as the function G(±1)=0.

It is characteristic that the numerical time *T* to obtain the balance decreases with the number *N* of nodes; $T\propto N^{-1/2}$[17]. As discussed there, this decrease was a consequence of Equation (2), where the rhs of the equation is expected to increase with *N*. (This mechanism is different from the compartmental model, where *T* increases with *N* [13]. For an elaborate analysis of this increase, see Appendix A therein). Also, the distance of the system from the final balanced state was found to decrease more sharply at the last stage of the process. As demonstrated in [17], this acceleration marks a high polarization of relations.

It is worthwhile to note that the jammed state of three subsets, mentioned at the end of the Introduction, is also absorbing when the evolution is driven by Equation (2). More generally, stable fixed points of continuous dynamics, where $x_{ij}\left(t\to \infty \right)=\pm 1$, coincide with stable solutions (including jammed states), obtained within the Constrained Triad Dynamics [13].

There are no parameters in Equation (2), except for the initial conditions, that is, the initial values of the relations $x_{ij}$. To verify this theory, some longitudinal data is needed. We used the matrix of relations between 34 members of some karate club, as recorded by Zachary [18]. The second time point is chosen after a conflict and the resulting partition into two groups, which is a balanced state. The content of the groups, with whom and against whom, can be obtained with Equation (2). In fact, the real partition was reproduced, with only one individual misplaced; in [14] it was erroneously stated that the partition obtained was exact.

## 4. The Riccati Equation

In [19], an analysis of the simplified version of Equation (2) is provided with G(x)=1. In matrix form, the equation can be written as $\dot{X}=X^{2}$, that is,

(3) $${\dot{x}}_{ij}=\sum_{k}x_{ik}x_{kj}$$

Marvel et al. [19] identify it as an example of the matrix Riccati Equation [19]. Although the solution expands to infinity in a finite time $t^{*}$, the authors show that slightly earlier ($t=t^{*}-\varepsilon$), the system reaches the balanced state in the signs of the matrix elements. Clearly, the final distribution of the signs cannot change anymore. There, $t^{*}=1/\lambda_{max}$, i.e., a reciprocal for the largest eigenvalue of X(0) (provided that it is positive). The analytical solution is first to diagonalize the matrix X(t=0), then to solve the equations for the eigenvalues $\lambda_{i}\left(t\right)=\lambda_{i}\left(0\right)/\left[1-\lambda_{i}\left(0\right)t\right]$, and then to reverse the diagonalization transformation to get

(4) $$X\left(t\right)=\frac{X(0)}{I-X(0)t}$$

For the purposes of this text, the following results of [19] are most interesting. First, for generic symmetric matrices X(0), the system tends to a balanced state. This is a confirmation of the numerical calculations in [14]. Second, consider μ to be the mean value of the entries $x_{ij}\left(t=0\right)$, randomly and independently selected from a symmetric distribution. If μ>0, the system evolves to “paradise”, with all the matrix elements of *X* positive. If μ≤0, the system splits into two parts, with positive links within each part and negative links between parts. In both cases, the balance appears, with a phase transition to paradise at μ=0.

The analytical results of [19] are also valuable as a significant step forward from a purely numerical approach [20,21], where the evolution of $x_{ij}$ stops when $x_{ij}=+1$ and ${\dot{x}}_{ij}>0$, or $x_{ij}=-1$ and ${\dot{x}}_{ij}<0$, for each pair (i,j). (For systems larger than N=100, numerical results of an integration of such equations of motion are less stable.) On the other hand, both Equations (2) and (3) lead the system to a (balanced) partition, which is most close to the initial (unbalanced) state of the network [22]. By “most close” we mean that the Hamming distance between the states is minimal.

Similarly to [13], Marvel et al. [19] also refer to an example of international relations, this time during World War II. There, data from Axelrod and Bennett [23] were used to construct the values of the relations with the formula $x_{ij}=propensity\left(i,j\right)\times size\left(i\right)\times size\left(j\right)$. With this tool, the actual structure of coalitions was reproduced, with two countries misplaced, Denmark and Portugal, out of seventeen.

The same Equation (3) has been used in [24] to explore the possibility of controlling the system by modifying the relations of one agent/node. An influence index has been proposed there: a minimal modification of links to get any desired balanced state. As expected, the US was found to be the most influential, with a large advantage over any other country. In addition, the authors observed “a global increase in the ability to achieve global harmony”. As we know today, this opportunity is wasted.

## 5. Asymmetric Relations

In the text above, we tacitly assumed that the relations are symmetric, i.e., $x_{ij}=x_{ji}$. However, multiple data indicate that context matters. Symmetry appears when we deal with the relation as the frequency of contacts, as was in the case of the karate club [18]. Symmetry does not appear if the data are collected with the question: Whom in your school class do you like most [21]?

Some care is needed in constructing the appropriate equation of motion. For the evolution of $x_{ij}$, we should distinguish between $x_{ik}x_{jk}$ and $x_{ik}x_{kj}$. The former choice is equivalent to the assumption that ${\dot{x}}_{ij}$ is the same as ${\dot{x}}_{ji}$, which is not our intention. Therefore, only the second version of the order of indices is correct.

Consider the equation of motion for a possibly asymmetric relation $x_{ij}$. Now, it should be supplemented with the equation for $x_{ji}$:

$$\begin{array}{c} {\dot{x}}_{ij}=\sum_{k}x_{ik}x_{kj}\\ {\dot{x}}_{ji}=\sum_{k}x_{jk}x_{ki} \end{array}$$

It is clear that the number of variables is doubled. In the simplest case N=3 (one triad), we have six equations. Remarkably, even such a small system provides new effects: for some initial values of the relations, we obtain limit cycles [20]. Some relations oscillate and remain positive, some relations oscillate and remain negative, some other relations oscillate and change sign.

While theoretically attractive, permanent oscillations of interpersonal relations would be boring for agents and, therefore, raise doubts. With this reservation in mind, the following equation of motion has been proposed in [21]:

(5) $${\dot{x}}_{ij}=\alpha \left(x_{ji}-x_{ij}\right)+\frac{1-\alpha }{N-2}\sum_{k}x_{ik}x_{kj}$$

The relations $x_{ij}$ are kept numerically within the range [−1,1]. Here, two processes compete. α is a coefficient that measures the amount of reciprocity, and 1−α is the term responsible for the tendency to balance. Under the effect of the first term, the symmetry of the relations is restored if only α is large enough. Our numerical experience shows that α=0.2 is enough to restore the symmetry. For α=1, the system divides into isolated pairs of agents.

In [21], Equation (5) has been used to discuss data on the relations between schoolchildren in 37 classroom groups in the Mexican City metropolitan area. The main result was that gender segregation is active in younger classes, of age less than 12 years. There, the identified groups are composed mostly of girls or mostly of boys. In older classes, these barriers are broken.

## 6. Classification of Fixed Points

Consider again the network of symmetric relations evolving in accordance with Equation (3). Setting $G\left(x\right)=1-x^{2}$ allows us to limit a priori the set of fixed points to those where $x_{ij}=\pm 1$. As mentioned above, the condition of stability is the negative sign of each diagonal element of the Jacobian. This means that for each link, $x_{ij}$ [25]

(6) $$x_{ij}\sum_{k}x_{ik}x_{kj}>0$$

It is worthwhile to limit our considerations to odd values of the number of nodes *N* to evade the situation where this expression is equal to zero.

The idea of a classification of fixed points is based on the assumption that the system contains some internally friendly and mutually hostile communities of $N_{i}$ nodes, where *i* is the community index. The stability conditions should allow one to obtain values of $N_{i}$, for which the related fixed point is stable.

Consider an example of three communities containing $N_{1}$, $N_{2}$, and $N_{3}$ nodes, respectively. The stability condition (6) of the above configuration for a positive link within $N_{1}$ is $\left(N_{1}-2\right)+N_{2}+N_{3}>0$. Further, the stability condition for a negative link between $N_{1}$ and $N_{2}$ is $N_{1}-1+N_{2}-1-N_{3}>0$. Obviously, many triples of numbers $\left(N_{1},N_{2},N_{3}\right)$ meet these criteria. In particular, the smallest jammed state (3,3,3), identified in [13], is stable. More generally, all jammed states (K,K,K) with odd values of *K* are also stable.

**Figure 2:**
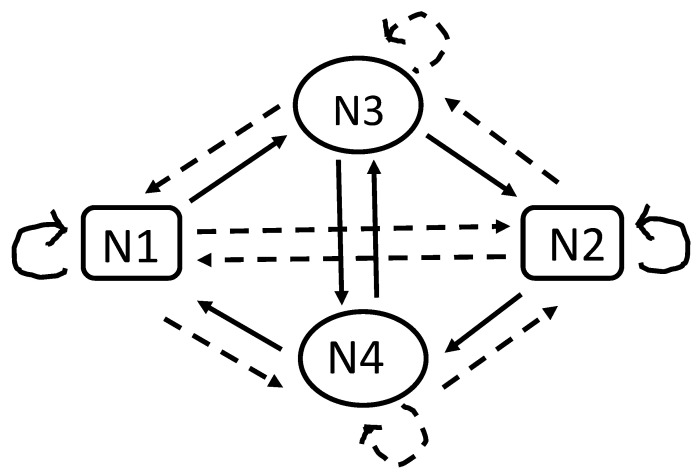
The generic scheme of asymmetric relations between four communities [26]. Continuous lines mean friendly relations, and dashed lines are hostile ones. Relations to members of the same community are marked by curvy lines, continuous for $N_{1}$ and $N_{2}$, and dashed for $N_{3}$ and $N_{4}$.

The same conceptual method was applied in [26] to asymmetric links, where $x_{ij}\neq x_{ji}$ is possible. The configurations of the matrices are more rich there. Consider the case of four communities $N_{i}$, i=1,2,3,4. A stable and apparently generic configuration is shown in Figure 2. There, a dashed arrow from $N_{a}$ to $N_{b}$ means a negative opinion ($x_{ab}$) of each individual from community *a* about each individual from community *b*. A self-directed link marks an evaluation of each member of a given community toward other members of the same community.

The stability condition of the exemplary configuration shown in Figure 2 is $N_{1}+N_{2}>N_{3}+N_{4}+2$. We can imagine a school class with two large and self-supporting groups, say, boys ($N_{1}$) and girls ($N_{2}$), mutually unfriendly. There could also be two maverick communities ($N_{3}$ and $N_{4}$) that do not accept others in the same group. Yet, those in $N_{3}$ accept those in $N_{4}$ and vice versa, as marked by two continuous vertical arrows in Figure 2. For example, $N_{3}$ could be two shy girls who dislike boys in $N_{1}$, who are accepted by all boys but rejected by other girls. And symmetrically: $\left(N_{4}\right)$ could represent a few of the boys, accepted by all girls but rejected by other boys and disliking each other. More examples of such structures can be found in [26], with some reference to belles-lettres.

In the paper [27], Equation (5) for ${\dot{x}}_{ij}$ has been supplemented by an additional term (with a coefficient γ), which measured the importance of the dynamics of ${\dot{x}}_{ji}$. A phase diagram has been obtained there, with three phases: balanced states, jammed states, and paradise.

## 7. Generalized Forces

In the paper [28] the potential energy is introduced together with its derivatives as generalized forces. The Dirichlet energy is defined [28] as

(7) $$D\left(x,\gamma \right)=\sum_{i>j}\gamma_{ij}{\left(x_{i}-x_{j}\right)}^{2}$$

where the link variable $\gamma_{ij}$ can be of any sign. If it is positive, positions $x_{i}$ and $x_{j}$ attract; otherwise, they repulse. The equation of motion is built with three additional conditions

(8) $$\frac{1}{2E}\sum_{i\neq j}\gamma_{ij}=Q>0$$

(9) $$\frac{1}{2E}\sum_{i\neq j}\gamma_{ij}^{2}=P$$

(10) $$\frac{1}{2E}\sum_{i\neq j}x_{i}^{2}=R$$

where E=N(N−1)/2 is the number of links. With the method of Lagrange multipliers, the related equation of motion for links is

(11) $${\dot{\gamma }}_{ij}=-\varepsilon \frac{\partial F}{\partial \gamma_{ij}}$$

where *F* is the free energy

(12) $$F=D-\frac{\mu }{2E}\sum_{i\neq j}\gamma_{ij}-\frac{\tau }{E}\sum_{i\neq j}\gamma_{ij}-\lambda \sum_{i\neq j}x_{i}^{2},$$

μ, τ and λ are Lagrange multipliers, and ε is an exogenous “stiffness” parameter. Additionally, nodes also evolve in a similar way:

(13) $${\dot{x}}_{i}=-\frac{\partial F}{\partial x_{i}}$$

Numerical calculations [28] have been performed for N=5, hence E=10. The numerical results show only two kinds of solution: a consensus state where all $x_{i}$ are positive and a balanced state with four individuals in one group and one individual in the second group. The latter option recalls the sociological effect of scapegoating [29], which has recently been discussed within another model [30]. Perhaps more solutions are possible for larger systems and/or for Q<0.

## 8. Government Formation

In [31], the evolution of the opinions of the members of parliament is discussed. The related equation of motion (Equation (7) in [31]) describes collective decision making:

(14) $$\dot{X}=-\Delta x+\pi A\psi \left(X\right)$$

where X is the vector of the opinions of MPs, A is the weighted adjacency matrix of the network with $a_{ij}$ positive between friends and negative between enemies, and Δ is a “damping” diagonal matrix of elements $\delta_{j}=\sum_{i\neq i}\left|a_{ij}\right|$. Further, π is an external scalar parameter, and ψ(X) is a vector of sigmoidal functions, saturated to ±1 in ±∞ and with the derivative equal to 1 at the origin $x_{i}=0$.

The main result of [31] is that the time required to form a government increases monotonically with initial frustration, the latter measured as the amount of imbalance. When the parameter π gets a critical value, a pitchfork bifurcation occurs in the opinions of the MPs, and this value also increases with system frustration. In other words, the greater the frustration (disorder) in the system, the larger the critical value of π to overcome it. These conclusions are supported with data on 29 European democracies, for elections between 1981 and 2019. Countries with systems qualified as presidential or semi-presidential (like France, Georgia, Poland, or Russia) were not considered.

Here, the bifurcation is equivalent to a partition of the network into two communities. It is legitimate to ask whether the obtained partition matches the actual partition of opinions of MPs. For this purpose, poll data should be collected about real opinions of politicians, at least in selected elections. Before such a procedure could be initialized, we can ask to what extent a balanced structure can be restored after being disturbed by some noise. This problem was investigated in [32,33] for fully connected graphs and for connected but sparse graphs. Namely, the equation of motion has been used

(15) $${\dot{x}}_{ij}=\Theta \left(1-x_{ij}\right)\Theta \left(1+x_{ij}\right)\sum_{k}\left(x_{ik}x_{kj}-\beta \right)$$

where $x_{ij}$ is a weighted symmetric link between nodes *i* and *j*, and β is a parameter. The value of β has been chosen in numerical experiments as follows:

- Add random numbers chosen from a homogeneous distribution from the range (−ε,+ε) to each matrix element $x_{ij}$;
- Apply Equation (15) to the disturbed matrix X with some trial value of β;
- In a series of such attempts, calculate the probability P(ε,β) of restoring the initial balanced structure;
- Modify β and repeat the procedure to get maximal *P*.

In this way, the best results on *P* have been obtained for β≈0.4. These results have been compared with the modularity maximization procedure by Newman and Girvan [34], and the results of Equation (15) were at least equivalent and sometimes better.

## 9. To Prevent the Balance

If the balance is interpreted as a conflict, as in [13], it is tempting to expand the theory with terms that could hamper polarization. One way is connected with layer structures. Multilayer networks as a research subject are known [35] to comprise several examples of social systems as an interplay between business and marriage networks in Renaissance Florence [36]. In [37], Equation (2) has been supplemented by an interlayer coupling,

(16) $${\dot{x}}_{ij}^{\alpha }=\left(1-{\left(x_{ij}^{\alpha }\right)}^{2}\right)\left(\beta^{\alpha }x_{ij}^{\alpha^{\prime }}+\frac{1}{N-2}\sum_{k=1}^{N}x_{ik}^{\alpha }x_{kj}^{\alpha }\right)$$

where $x_{ij}^{\alpha }$ is a weighted link between nodes *i* and *j* in the α layer, α=1,2, $\alpha^{\prime }=3-\alpha$, and $\beta_{\alpha }$ is a measure of an influence of links of the $\alpha^{\prime }$ layer on links of the α layer. The coefficients $\beta_{1}$ and $\beta_{2}$ can be different. In particular, if both $\beta_{1}$ and $\beta_{2}$ are large and $\beta_{1}\beta_{2}<0$, oscillations of the coefficients $x_{ij}^{\alpha }$ can be observed. This case of strong asymmetry is reminiscent of the case of asymmetry of links $x_{ij}\neq x_{ji}$[20], where oscillations of links have been observed as well. Apparently, the asymmetry produces a kind of negative feedback, as in harmonic oscillator where $\dot{x}=+v$ and $\dot{v}=-x$.

In [37], two examples are mentioned of a situation where one layer is dominated by another layer, for example, $|\beta_{1}\left|>>\right|\beta_{2}|$. In the first example, the first layer relates to the roles of football players during a match, and the second layer relates to a solidarity based on ethnic bonds. Strangely, the ball would be passed only between players from the same country. In the second example, there are two economic strategies, each consisting of a series of statements. Although all these statements could be mutually consistent, often they cease to be so when presented to public opinion. If the latter dominates, only one strategy can be adopted.

Polarization can also be reduced when individual attributes of nodes are taken into account. In [38], Equation (16) is rewritten by replacing the interlayer coupling with a term dependent on the attributes of the nodes. Although for our purposes the direct mathematical form of this term is of secondary importance, its inclusion can be a method to take into account the homophily principle: like attracts like [39]. As a rule, positive signs of this additional term destabilize negative relations and therefore reduce the conflict.

## 10. Peace and War

In [40], the following equation of motion for symmetric relations $x_{ij}$ has been proposed:

(17) $${\dot{x}}_{ij}=-\beta \left(x_{ij}-x_{Dij}\right)+L\tanh\left(\frac{1}{\gamma }\sum_{k}x_{ik}x_{kj}\right)+x_{Tij}$$

where β, *L* and γ are scalar parameters that design the mutual validity of particular terms, the matrix elements $x_{Dij}$ are approximately equal to the stationary values of $x_{ij}$ if the term with β dominates, and the matrix $x_{Tij}$ represents noise; the latter is omitted in the discussion below. In the term proportional to *L*, the function tanh is helpful to keep the relations $x_{ij}$ finite. Close to the origin, this term is proportional to the standard expression $\sum_{k}x_{ik}x_{kj}$. Typically $x_{ij}\left(t=0\right)=x_{Dij}$.

In the text, an interpretation of the approximate stationary values of $x_{ij}$ is proposed in terms of the phases of international relations. From large positive to large negative values, the phases are as follows: allies in a war, offense pact with extensive or modest military obligations, allies in defense or non-aggression pact, neutral, rivals in hostile relations, rivals with threat to use force, display of force, use of force, and rivals in a war. It appears that the actual phase depends on the parameters β, *L* and γ. In particular, a sharp bifurcation is observed from peace to war when *L* grows, a bistable solution of war and peace, and a hysteresis effect, the transition from war to peace, occurs at a different value of L/γ than the transition from peace to war. These transitions appear as distinct changes in the values of $x_{ij}$. Let us add that the size relations $|x_{ij}|$ in war are clearly larger than in peace.

The special case of two equal opposite factions is also analyzed [40]. In this case, Equation (17) is reduced to a set of two equations, numbered by an index ϕ:

(18) $$\dot{x}=-\beta \left(x-\phi \mu \right)+\phi L\tanh\left(\frac{N}{\gamma }x^{2}\right)$$

where ϕ=±1 for the in-group and out-group relations, respectively.

In subsequent parts of the paper [40], the results of the analysis have been applied to discuss the case of World War I. Five powers were considered according to the situation in 1913: the United Kingdom, France, and Russia in one faction and Germany and Austria-Hungary in the other. (The contribution of Italy was treated as a noise.) The course of simulations was commented on with a detailed knowledge of the historical course of events.

One year later, a similar approach was formulated in [41]. In this case, the equation of motion was as follows:

(19) $${\dot{x}}_{ij}=-\beta x_{ij}+A\sum_{k}\tanh\left(x_{ik}/\alpha \right)\tanh\left(x_{kj}/\alpha \right)$$

with constant parameters β, *A*, and α. The results highlight the role of the group size *N* in a transition from neutral to bipolar states of the network.

## 11. Moral Values

The concept of Heider balance can also be applied to beliefs, and the initial values of the relations between beliefs can be introduced as correlations between the numbers of believers. Further, the time evolution of these relations is determined from Equation (2), until the balance state is attained. In this way, communities of beliefs can be identified, related to groups of individuals who adhered to those beliefs.

Our first example relates to longitudinal data collected by polls in 44 US states between 1974 and 1988 [42]. The list of nine issues comprised tolerance, antiracism, abortion, religiosity, homosexuality, public feminism, environment spending, welfare spending, and the death penalty. Data on support for these issues allowed us to calculate the 9×9 matrix of Pearson correlations r(i,j),

(20) $$r\left(i,j\right)=\frac{\sum_{s}\left(a\left(s,i\right)-\bar{a}\left(i\right)\right)\left(a\left(s,j\right)-\bar{a}\left(j\right)\right)}{\sqrt{\sum_{s}{\left(a\left(s,i\right)-\bar{a}\left(i\right)\right)}^{2}}\sqrt{\sum_{s}{\left(a\left(s,j\right)-\bar{a}\left(j\right)\right)}^{2}}}$$

where a(s,i) is the support for issue *i* in state *s*, averaged over years, and $\bar{a}\left(i\right)$ is the support for issue *i*, averaged over states. The numbers r(i,j) serve as initial values of matrix elements $x_{ij}$ for Equation (2). As a result, the time evolution produced two sets of issues: religiosity and welfare spending in one set, and all remaining seven issues in another set [22]. This partition conforms with the so-called Comfort Hypothesis by Earl Babbie, a leading figure in sociology. The hypothesis states: “Parishioners whose life situations most deprive them of satisfaction and fulfillment in the secular society turn to the church for comfort and substitute rewards” [43]. In other words, those who expect help from the state also expect help from the heavens.

The same research strategy has been applied to the data on life aims, collected in three rounds in 2012, 2014, and 2016 in the framework of the Polish European Social Survey [44]. Again, the results allow one to distinguish two groups which, after [45], are called Defenders and Explorers. The typical position of a Defender is to live in secure and safe surroundings, to follow traditions and customs, to behave properly, to do what is told and follow rules, to be humble and modest, not draw attention, and to be respected by others [46]. In contrast, the list of life aims of an Explorer contains the following: to try new and different things in life, to make one’s own decisions and be free, to seek fun and things that give pleasure, to be rich and have money and expensive things, to seek adventures and have an exciting life, to think new ideas and be creative [46], etc. Of the 21 aims, 4 belong to Defenders or Explorers in different rounds. It is worthwhile to add that although the formulation of the issues and a significant partition depends on the particular country and/or culture, the very appearance of the categories “Defenders” and “Explorers” seems to be universal.

## 12. Summary

The main thread of this mini-review is the evolution of the term containing $x_{ik}x_{kj}$ in the equation of motion for $x_{ij}$. Technically, subsequent models provide more and more refined methods to evade the unlimited growth over time of the relation $x_{ij}$, which appears in the analytical solution of ${\dot{x}}_{ij}=\sum_{k}x_{ik}x_{kj}$ [19]. From a purely numerical condition $-1<x_{ij}<1$, next to using the prefactor $1-x^{2}$, next to an application of the function tanh(.) in different variants together with relaxation terms such as −βx, these methods lead to different results of the stability analysis.

In parallel, new characteristics of the Heider balance problem have appeared: possible asymmetry of the relations ($x_{ij}\neq x_{ji}$), analysis of the jammed states, and different topologies of the networks considered. In early formulations, the models dealt with complete graphs: even if initially equal to zero, each link continued to evolve towards a positive or negative value if only the network was initially connected. It was taken into account that the network topology indicated that some links are absent initially and during the time evolution. With this assumption, more effort has been put into Monte Carlo methods, which, however, remain outside the scope of this text.

All the texts included in this short review reveal some interest in social applications. We hope that the text can be useful as a component of a survey of the literature on Heider or structural balance in social phenomena.
